# Uptake of human papillomavirus (HPV) vaccination in Hong Kong: Facilitators and barriers among adolescent girls and their parents

**DOI:** 10.1371/journal.pone.0194159

**Published:** 2018-03-15

**Authors:** Winnie Wing Yan Yuen, Albert Lee, Paul K. S. Chan, Lynn Tran, Erica Sayko

**Affiliations:** 1 Karen Leung Foundation Limited, Hong Kong SAR, China; 2 Jockey Club School of Public Health and Primary Care, The Chinese University of Hong Kong, Prince of Wales Hospital, Shatin, New Territories, Hong Kong SAR, China; 3 Department of Microbiology, Faculty of Medicine, The Chinese University of Hong Kong, Prince of Wales Hospital, Shatin, New Territories, Hong Kong SAR, China; Rudjer Boskovic Institute, CROATIA

## Abstract

The present study is aimed at assessing the feasibility of delivering the HPV (human papillomavirus) vaccine to girls through a school-based program in Hong Kong, as well as to examine the facilitators and barriers associated with their participation. We approached 1,229 eligible girls aged 9 to 14 at eight schools in Hong Kong to join the program and then delivered the bivalent HPV vaccine at 0 and 6 months over the course of one school year. The students and their parents completed separate questionnaires to indicate their decision on whether or not to participate, and to assess their knowledge of cervical cancer and the HPV vaccine. The overall vaccine uptake was 81.4% (1,000/1,229) for the first dose and 80.8% (993/1,229) for the second dose. Parents and students were given separate questionnaires and asked whether or not they would like to participate in the vaccination program. 87.1% (1,010/1,160) of parents and 84.9% (974/1,147) of students indicated that they would join the program. The reasons associated with parents’ decision not to vaccinate their daughters primarily included concerns around side effects and safety. Multivariate regression analysis showed that parents who thought that the vaccine would protect their daughter from getting cervical cancer (OR = 3.16, 95% CI = 1.39–7.15, *p* < .01), and those who reported having a doctor’s recommendation (OR = 4.54, 95% CI = 1.05–19.57, p < .05) were more likely to join the program. In contrast, parents who had never heard of the vaccine (OR = .15, 95% CI = .03–.71, *p* < .02), those who were willing to pay more than HK$2,000 for the vaccine (OR = .39, 95% CI = .19–.81, *p* < .05), or had a preference to access it through a private clinic (OR = .44, 95% CI = .26–.75, *p* < .01) were significantly less likely to allow their daughter to join the program. Delivery of the HPV vaccine with high uptake rate in a school setting is feasible in Hong Kong. Engaging key stakeholders including school administrators, teachers and community physicians, and providing relevant information on safety and vaccine effectiveness to parents were important to the success of the program.

## Introduction

Vaccination against human papillomavirus (HPV) has the potential to drastically reduce the incidence of cervical cancer given that approximately 70% of cervical cancers are caused by HPV-16 or HPV-18 (NACI, 2015). Vaccination is especially important in regions such as Hong Kong where a comprehensive cervical screening program was conceived in 2004 but never effectively implemented to this date. In the most recent survey conducted by the Department of Health of Hong Kong [1], just over half (57%) of women surveyed had a cervical smear in the last 3 years. Unlike many Western countries, Hong Kong has not yet implemented a universal HPV vaccination program and the current uptake rate remains low; only 7–9% of school-aged girls [2–4] and 9.7% of university aged girls women [5] are reported to be vaccinated. Additionally, adolescent girls [4], university-aged women [5], and mothers of school-aged daughters [2] reported that the cost of the vaccination is a barrier to vaccination. Lack of awareness as well as lack of access to information about the vaccine and where to receive it have also been reported as barriers [4]. A universal school-based vaccination program that offers the vaccine free of charge is proposed as part of a comprehensive cervical cancer prevention strategy. Students in Hong Kong have expressed the desire for schools to provide more information on cervical cancer prevention [3]. A school-based approach will likely boost uptake rates as it can potentially increase students’ and parents’ knowledge, ensure improved access and coverage and address other barriers such as concerns about safety and cost. The main objective of this study was to investigate the feasibility and acceptability of a universal school-based HPV vaccination program.

Our methodology relies on the health belief model and theories [6–8], which emphasize that a person’s perception, such as perception of disease severity, susceptibility, barriers and benefits, and social norms influence the intention and actual health seeking behavior. The Vaccine Perceptions, Accountability and Adherence Model for HPV vaccine developed by Katz et al. [9] highlights key factors in vaccine acceptance and adherence which include both structural and sociocultural factors, and stress the importance of both caregiver and adolescent perception such as health beliefs and knowledge. As an example, in low-income settings, structural barriers such as cost of the vaccine may supersede other factors such as perceptions of vaccine effectiveness and impact uptake. The health belief model and this HPV conceptual framework informed the design of our study, focusing on the uptake rate of the vaccine as the main outcome as well as investigating social, structural, and environmental factors that act as barriers or facilitators. The aim of this study was to examine whether or not these factors would influence parents’ and students’ acceptance and uptake of the HPV vaccine.

A series of studies conducted among school-aged Chinese girls in Hong Kong showed that the factors associated with the intention to be vaccinated included perceived severity of HPV infection, safety and efficacy of the vaccine, and recommendation by general practitioners or significant others [3, 10]. In addition, a systematic review also showed that obtaining reliable information from health care providers was related to a higher uptake rate in a school-based HPV vaccination program [11]. However, as most of the literature examined the association between perception and intention, we sought to explore the factors affecting actual behavior from both students and parents through this study.

The main objective of this study was to determine whether a school-based program for HPV vaccination was feasible in Hong Kong, based on uptake rate among a sample of adolescent, female students. Additionally, we sought to better understand reasons that contribute to both students’ and parents’ decision to participate in a school-based HPV vaccination program. Finally, recommendations were made based on our findings for other schools, administrators, charities and policymakers who may be considering implementation of HPV vaccination programs in Hong Kong.

## Materials and methods

### Study design

The study was part of a school-based HPV vaccination and intervention program conducted in Hong Kong between May 2015 and July 2016. The participants were female students aged nine to 14 and enrolled in primary grades four to six and secondary grades one to two. Invitations were sent to public and government-funded schools in Tuen Mun and Yuen Long, two districts in New Territory West. These schools previously participated in other health promotion programs organized by the Centre for Health Education and Health Promotion at The Chinese University of Hong Kong (CUHK). The inclusion criteria were: (1) girls between the ages of 9 to 14; and (2) the ability to read and write in Chinese (both students and parents) (3) girls who had not been previously vaccinated for HPV. The program included an education component and surveys that were distributed to both parents and students. The surveys were based on the Health Belief Model [6, 8] and the Theory of Reasoned Action [7] and the one used in previous school based HPV programs [3, 12] to assess the potential determinants leading to their uptake of the HPV vaccine.

### Ethics approval

Written consent from parents was obtained as a pre-requisite to their daughter joining the vaccination program. Ethics approval for this study was obtained from the Joint Chinese University of Hong Kong-New Territories East Cluster Clinical Research Ethics Committee (2014-493T).

### Procedures

Eight schools (six primary schools and two secondary schools) agreed to participate in the program. A letter of invitation was sent to the parents of all eligible students to inform them about the school-based HPV vaccination program and the research nature of the program. Written parental consent was obtained for girls to join the program as well as to complete the questionnaire.

After obtaining parental consent, students were asked to watch two educational videos and provided with educational materials to take home to their parents. The educational materials and videos were also posted on a website and made available online for parents to view. Students and their parents were asked to complete a self-administered questionnaire that was developed based on previous studies [3, 12, 13] and pilot tested. Questions included knowledge and attitudes towards cervical cancer, HPV prevention and vaccination, as well as demographic information.

A team of research staff, nurses, physicians and volunteers carried out delivery of the school-based vaccination program. A research coordinator and nurse worked with the schools to respond to any questions that parents had about the vaccine. Under the supervision of a primary care doctor, a nurse administered the first dose of the HPV vaccine to all eligible students. Medical staff assessed any students who reported feeling unwell after vaccination and no adverse outcomes were reported. After the first dose, students were given a vaccination card and reminded that they would receive the second dose six months later. Research staff coordinated the delivery of the second vaccination dose with the schools. The second vaccination doses were also provided on-site at the schools and delivered by a nurse under the supervision of a doctor. Students who were unable to obtain their second vaccination dose at school were able to obtain it through a designated family physician in the community. Prior to launch, the program was piloted with four schools in the same district to finalize logistics.

### Measures

Two sets of self-administered questionnaires were developed with reference to previous studies in Hong Kong. These were used in this study to obtain information from parents and students. The questionnaires were designed to measure factors that might be associated with the uptake of HPV vaccine, including awareness and knowledge of cervical cancer, HPV infection and HPV vaccine, perceived impact of the vaccine, attitudes and beliefs about cervical cancer prevention, as well as other socio-demographic information.

There were altogether 26 items in the parents’ questionnaire. First, parents were asked to indicate whether their daughter had been given permission to join the school-based HPV vaccination program. Next, it explored reasons for their decisions, such as whether or not they thought the vaccine was safe, whether the vaccine was recommended to them by a doctor, and if they feared possible side effects. Parents were then asked to respond to statements on cervical cancer, such as, “cervical cancer is caused by viral infection” and “females with multiple sex partners have a higher risk of getting cervical cancer.” They had the option to answer “yes”, “no” or “don’t know”. Each correct answer received 1 point while an incorrect answer or “don’t know” received 0 points. Other questions included knowledge of the HPV vaccine such as whether it was able to “prevent all sexually transmitted infections” and if it would “not affect growth”. Parents’ health seeking behaviors and demographic information including age, education level, marital status, and monthly family income were also collected.

There were 15 questions in the students’ questionnaire. Similar to the parents’ version, they were first asked to indicate whether they had joined the program and then the reasons impacting their decision. There were questions tapping into their knowledge of cervical cancer, e.g. “cervical cancer is one of the common cancers found in women in Hong Kong” and the HPV vaccine, such as “girls should not receive HPV vaccine before their first menstruation”, as well as their information sources for this knowledge. Both questionnaires were refined by experts in public health and infectious diseases and then tested for face and content validation in the four pilot schools.

### Statistical analysis

The denominator for calculating the uptake rate was the number of eligible girls (between the ages of 9 to 14) in participating schools. The difference between the uptake rate among primary and secondary schools was calculated using *chi*-square test. In addition, responses of parents and students who chose to join or not join the program were compared using chi-square test or Fisher’s exact test. Univariate and multivariate logistic regressions were performed to investigate whether knowledge, attitudes and perception of cervical cancer and HPV vaccine were associated with participation in the program. Variables with significant level less than 0.2 in the univariate regression analyses were included in the multivariate logistic regressions.

## Results

### Uptake of the HPV vaccine

**Fig 1:**
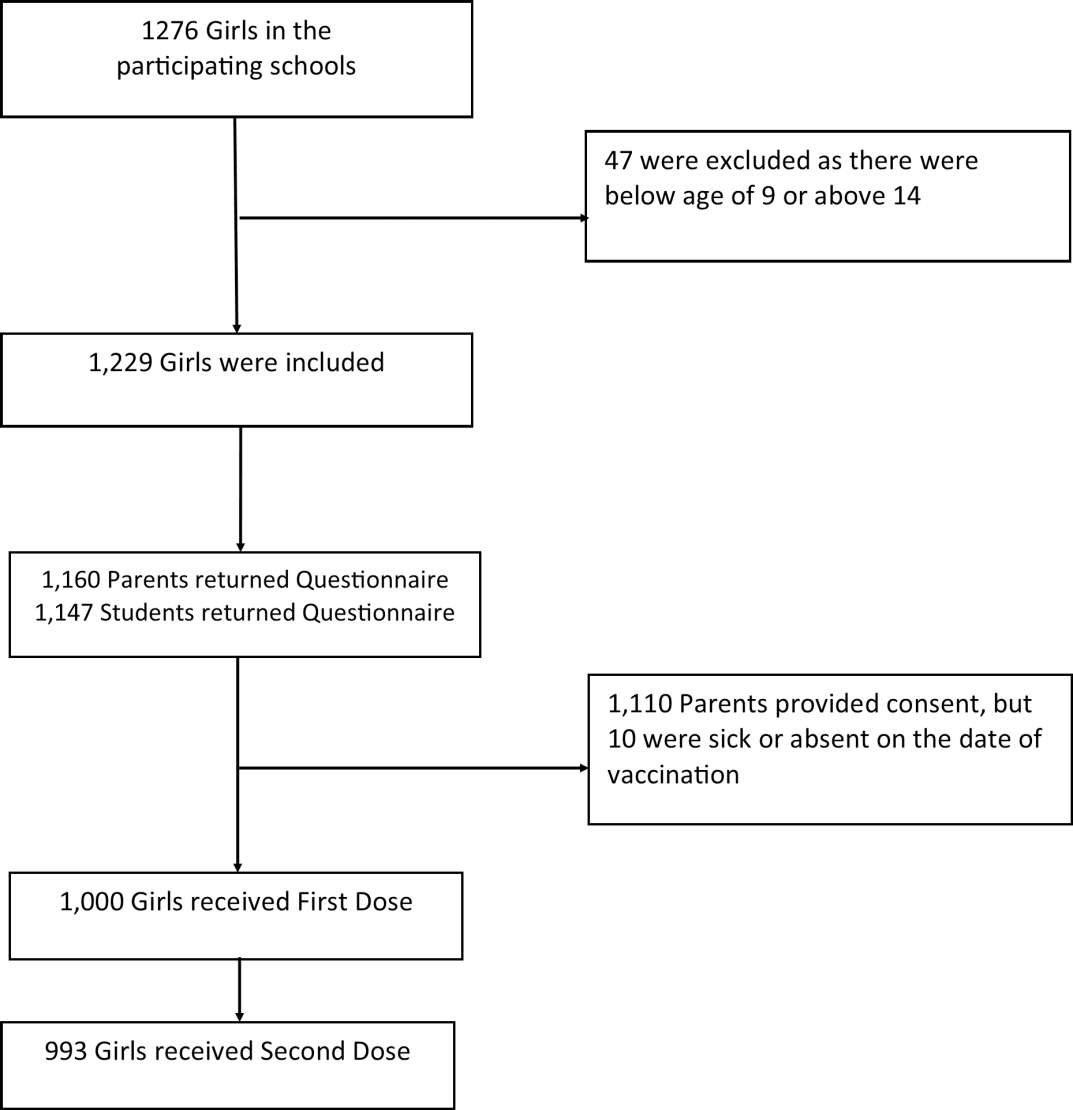
Flow chart—HPV vaccination program and research study.

There were 1,276 girls enrolled at the eight participating schools, of which only 1,229 girls met the inclusion criteria. In total, 1,000 girls received the first dose. Average uptake among the schools was 81.4%. 993 girls received the second dose and average uptake was 80.8%. Of the 1,000 girls who received the first dose, 80.2% came from the six primary schools whereas the remaining 19.8% were from the two secondary schools. The uptake rate was 83.6% among primary school girls and 70.4% among secondary school girls. Chi-square test showed that there was a significant difference in the uptake rate between primary and secondary school girls (χ^2^ = 16.93, p>0.001). No adverse events were reported in the program. Fig 1 is a flow chart of this study.

Parents or guardians of the girls were also invited to answer a questionnaire and 1,160 responses were collected (90.9%). The demographics of the parents who completed the questionnaire are summarized in Table 1.

**Table 1:** Demographics of the parents as compared to the statistics from the Hong Kong census and statistics department [14].

	*n*	%	% from census
**Gender**			
Male	123	11.0	NA
Female	998	89.0	NA
**Relationship**			
Parents	1066	94.8	NA
Relatives	33	3.0	NA
Guardian / others	25	2.2	NA
**Age**			
Below 30	27	2.4	NA
31–40	512	45.9	NA
41–50	495	44.4	NA
51 or above	81	7.3	NA
**Monthly household income (HK$)**			
Below 10,000	197	18.1	20.4
10,000 to <20,000	433	39.6	21.9
20,000 to < 30,000	208	19.0	17.6
30,000 or above	256	23.4	40.0
**Education level**			
Primary or below	118	10.6	19.6
Secondary	885	79.8	50.5
Tertiary or above	106	9.6	29.8

Of the parents who submitted the questionnaire, 1,010 provided consent for their daughters to be vaccinated through the school-based program. Twenty-seven respondents had previously vaccinated their daughters elsewhere. Among those who chose to vaccinate their daughters, over 70% indicated that they believed the vaccine would provide effective prevention of cervical cancer and reported this factor as influential in their decision-making. In addition, over 71% indicated that the safety of the vaccine was an influential or extremely influential factor. Furthermore, not having to pay for the vaccine was also reported as an influential factor with approximately 62% of parents reporting it as being an influential or extremely influential factor in their decision-making (Table 2).

**Table 2:** Factors reported by parents to be influential in their decision-making.

	Level of influence			
	Not at all influential *n* (%)	Not influential *n* (%)	Influential *n* (%)	Extremely influential *n* (%)
**Factors for joining the program**				
Program is organized by school	168 (17.3)	316 (32.6)	370 (38.1)	116 (12.0)
Program is recommended by the university	154 (16.2)	373 (39.1)	328 (34.4)	98 (10.3)
Effective prevention of cervical cancer	99 (10.2)	150 (15.5)	417 (43.1)	302 (31.2)
HPV vaccine is safe	97 (10.4)	171 (18.3)	412 (44.0)	256 (27.3)
Vaccination is free of charge	131 (13.9)	232 (24.5)	380 (40.2)	202 (21.4)
HPV vaccine is recommended by doctor(s)	125 (13.5)	290 (31.4)	388 (42.0)	121 (13.1)
**Factors for not joining the program**				
Fear of possible side effects	11 (8.7)	27 (21.3)	66 (52.0)	23 (18.1)
Doubt the effectiveness of vaccine	11 (8.9)	55 (44.7)	48 (39.0)	9 (7.3)
Lacking doctor’s recommendation	16 (13.4)	52 (43.7)	47 (39.5)	4 (3.4)
Lacking government’s recommendation	13 (11.0)	68 (57.6)	34 (28.8)	3 (2.6)
Worried others might think daughter is sexually active	41 (34.5)	66 (55.5)	11 (9.2)	1 (0.8)
Worried that daughter will start having sex earlier if vaccinated	42 (35.0)	63 (52.5)	13 (10.8)	2 (1.7)

One hundred and twenty-three parents or guardians decided not to vaccinate their daughters for HPV. Among those who declined, 18.1% and 52.0% indicated that fear of side effects was an extremely influential or influential factor in their decision-making, and approximately 46% reported that they did not think the vaccine was effective. Few parents indicated that they were worried about the perceived perception of promiscuity (others thinking their daughter was sexually active) as a barrier. Over half (64.5%) of all parents indicated that they would be willing to pay less than US$125 for the entire course of the HPV vaccination.

The 1,276 girls invited to take part in the program were also asked to complete a survey. A total of 1,147 girls (89.9%) returned the completed questionnaire, with 895 responses from primary school girls and 252 from secondary school girls. There were 974 students who indicated that they would join the program, specific factors that students rated as highly influential in their decision to be vaccinated included the belief that the vaccine could effectively prevent cervical cancer (32.1%), that it was safe (26.1%), and that it was free of cost (23.8%). One hundred and thirty-five girls reported they did not participate in the vaccination program. Of those, 15.9% reported that objection from their parents or a family member was a significant factor that influenced whether or not they would join the program. Fear of possible side effects was also a consideration with 10.5% and 33.9% indicating that it was an extremely influential or influential factor for them respectively. It is also interesting to note that about 33% of girls rated fear of pain to be an influential factor for not participating in the program (Table 3).

**Table 3:** Factors reported by students to be influential in their decision-making.

	Level of influence			
	Not at all influential *n* (%)	Not influential *n* (%)	Influential *n* (%)	Extremely influential *n* (%)
**Factors for joining the program (*n* = 974)**				
This program is organized by school	265 (27.7)	319 (33.4)	286 (29.9)	86 (9)
This program is recommended by university	245 (26.1)	397 (42.3)	240 (25.6)	57 (6.1)
Vaccine can effectively prevent cervical cancer	162 (16.8)	143 (14.8)	350 (36.3)	310 (32.1)
Vaccine is safe	157 (16.7)	180 (19.1)	358 (38.0)	246 (26.1)
Free vaccination	216 (22.8%)	209 (22.1)	297 (31.4)	225 (23.8)
Doctor’s recommendation	239 (25.7)	315 (33.8)	269 (28.9)	108 (11.6)
Parents’ or family members’ recommendation	194 (20.6)	257 (27.2)	327 (34.6)	166 (17.6)
Classmates/friends join the program	271 (28.9)	320 (34.2)	267 (28.5)	79 (8.4)
**Factors for not joining the program (*n* = 135)**				
Possible side effects	24 (19.4)	45 (36.3)	42 (33.9)	13 (10.5)
Doubt the effectiveness of vaccine	26 (21.1)	54 (43.9)	35 (28.5)	8 (6.5)
Absence of doctor’s recommendation	31 (25.0)	58 (46.8)	29 (23.4)	6 (4.8)
Absence of government recommendation	35 (28.7)	68 (55.7)	15 (12.3)	4 (3.3)
Objection from parents/family members	18 (14.3)	43 (34.1)	45 (35.7)	20 (15.9)
Classmates/friends have not joined	42 (34.7)	54 (44.6)	20 (16.5)	5 (4.1)
Worry that vaccine may affect their growth	27 (2.4)	59 (48.4)	26 (21.3)	10 (8.2)
Fear of pain from injection	29 (23.8)	53 (43.4)	25 (20.5)	15 (12.3)

### Participation factors among parents

The responses from parents who allowed their daughters to join the HPV vaccination program and those who did not allow their daughters to join the program are compared in Table 4. A higher percentage of parents in the “joining” group (98.8%) compared to the “not joining” group (95.8%) indicated that they had heard about HPV vaccination in the past. Also, a significantly higher proportion of the “joining” group of parents knew the correct statements relating to cervical cancer, for example that it is treatable at an early stage. When compared to the parents in the “not joining” group, a higher percentage of parents in the “joining” group indicated that the HPV vaccine would not affect their daughter’s growth (62.7% vs. 78.9%), getting cervical cancer would affect their daughter tremendously (89.7% vs. 95.3%), the HPV vaccine could protect their daughter from cervical cancer (81.3% vs. 94.3%), and that they had sufficient knowledge of the HPV vaccine (48% vs. 63.5%). However, they did not perceive that they had enough information from doctors or experts (50.8%) as compared to their counterparts (65.3%). In terms of demographics, a higher percentage of families who chose to join the program had a monthly income of less than HK$30,000. A greater proportion of parents in the “joining” group reported that they would pay less than HK$2,000 for the HPV vaccine.

**Table 4:** Comparison between parents who allowed their children to join and not join the HPV vaccination program (univariate regressions).

	*Joined the program*		OR	95% CI	*p*
	No *n* (%)	Yes *n* (%)			
**HPV vaccine information source (*n* = 1,118)**					
Doctor	19 (15.8%)	199 (19.9%)	1.32	.79–2.21	.29
School	45 (37.5%)	360 (36.1%)	0.94	.64–1.39	.94
Poster	70 (58.3%)	571 (57.1%)	0.96	.65–1.40	.41
Media	93 (77.5%)	805 (80.7%)	1.21	.77–1.91	.41
Friends / relatives	20 (16.7%)	185 (18.5%)	1.14	.69–1.89	.62
Never heard of HPV	5 (4.2%)	12 (1.2%)	0.28	.10 –.81	0.02*
**Correct cervical cancer knowledge**			1.09	.99–1.21	.08
Cervical cancer is common (*n* = 1,060)	84 (80.8%)	854 (89.3%)	1.99	1.17–3.38	.01*
Early stage cervical cancer is treatable (*n* = 1,060)	83 (79.0%)	830 (86.9%)	1.76	1.06–2.92	.03*
Cervical cancer may affect fertility (*n* = 1,057)	81 (77.1%)	786 (82.6%)	1.40	.86–2.28	.17
Increase risk with multiple sex partners (*n* = 1,055)	76 (72.4%)	701 (73.8%)	1.07	.68–1.69	.76
Women have a fair chance of contracting HPV (*n* = 1,055)	53 (50.5%)	502 (52.8%)	1.10	.74–1.65	.65
Pap smears can prevent cervical cancer (*n* = 1,056)	61 (58.7%)	616 (64.8%)	1.30	.86–1.96	.21
Cervical cancer is caused by viral infection (*n* = 1,055)	76 (73.1%)	625 (65.7%)	.71	.45–1.11	.13
A woman dies of cervical cancer every 3 days (*n* = 1,056)	33 (32.0%)	448 (47.0%)	1.88	1.22–2.90	.004**
**Correct symptoms knowledge (*n* = 1,026)**			.91	.73–1.14	.41
Waist pain	88 (88.9%)	748 (80.7%)	.52	.27–1.00	0.05
Fear of cold	96 (97.0%)	874 (94.3%)	.52	.16–1.68	.52
Abnormal bleeding between menses	79 (79.8%)	726 (78.3%)	.91	.55–1.53	.73
Breast pain	89 (89.9%)	810 (87.4%)	.78	.39–1.54	.47
Bleeding after sexual intercourse	64 (64.6%)	658 (71.0%)	1.34	.87–2.07	.19
**Correct HPV vaccine knowledge (*n* = 1,042)**			1.45	.92–2.27	.11
Should not receive before their first menstruation	92 (90.2%)	867 (92.2%)	1.29	.64–2.59	.47
Ideal vaccination time is before the first time having sex	70 (68.6%)	615 (65.4%)	.87	.56–1.34	.52
HPV vaccination can prevent all STIs	96 (94.1%)	842 (89.6%)	.54	.23–1.26	.15
HPV vaccination would not affect growth	64 (62.7%)	742 (78.9%)	2.23	1.45–3.42	< .001***
Females who are sexually active can still receive HPV vaccine to prevent future infection	61 (59.8%)	645 (68.6%)	1.17	.98–1.40	.09
**Likelihood**					
My daughter will be infected by HPV (*n* = 1,013)	49 (52.1%)	435 (47.3%)	.83	.54–1.26	.38
My daughter will get cervical cancer (*n* = 1,011)	44 (47.8%)	448 (48.7%)	1.04	.68–1.60	.87
Getting cervical cancer will affect my daughter tremendously (*n* = 1,027)	87 (89.7%)	886 (95.3%)	2.32	1.13–4.76	.02
HPV vaccine protects my daughter from HPV infection (*n* = 1,039)	78 (81.3%)	889 (94.3%)	3.80	2.12–6.80	< .001***
Perceived sufficient understanding of HPV vaccine (*n* = 1,055)	27 (26.2%)	213 (22.4%)	1.23	.77–1.96	.38
Doctor recommended before (*n* = 1,056)	4 (3.8%)	106 (11.1%)	3.17	1.43–8.78	.03*
Has regular family doctor (*n* = 1,077)	51 (47.7%)	352 (36.3%)	.63	.42 –.93	.02*
Received all vaccines at Department of Health (*n* = 1,061)	74 (74.0%)	728 (75.8%)	1.10	.69–1.76	.70
Mother had a pap smear in the past 3 years (*n* = 1,062)	65 (61.3%)	537 (56.2%)	0.81	.54–1.22	.31
Mother received HPV vaccination (*n* = 1,061)	10 (9.7%)	94 (9.8%)	1.01	.51–2.01	.97
Government should provide HPV vaccine to boys & girls (*n* = 1,052)	38 (37.6%)	312 (32.8%)	.81	.53–1.24	.33
Willing to pay more than HK$2,000 for HPV vaccine (*n* = 1,046)	18 (18.8%)	76 (8.0%)	.38	.21 –.66	.001**
Preferred location—maternal and child health centers (*n* = 1,057)	64 (62.1%)	580 (60.8%)	.95	.62–1.44	.79
Preferred location—private clinics (*n* = 1,057)	48 (46.6%)	295 (30.9)	.51	.34 –.77	.001**
Preferred location–schools (*n* = 1,057)	48 (46.6%)	561 (58.8%)	1.64	1.09–2.46	.02*
Watched educational video (*n* = 1,056)	20 (19.4%)	232 (24.3%)	1.34	.80–2.22	.27
**Gender (*n* = 1,076)–**female	99 (93.4%)	858 (88.5%)	.54	.25–1.20	.13
**Age (*n* = 1,070)–**above 40	61 (58.7%)	495 (51.2%)	.74	.49–1.12	.15
**Education level (*n* = 1,065)–**secondary or above	96 (92.3%)	861 (89.6%)	.72	.34–1.52	.39
**Monthly household income (*n* = 1,053)—**above HK$30,000	33 (32.7%)	216 (22.7%)	.61	.39 –.94	.03

* = significant at *p*

** = significant at *p*< .01.

*** = significant at *p*

Tables 4 and 5 also illustrate the factors associated with parents’ decision to allow their daughters to join the vaccination program. Univariate logistic regression revealed that having correct knowledge of cervical cancer (i.e. a woman dies every 3 days; 47.0% vs 32.0%, OR = 1.88, 95% CI = 1.22–2.90, *p* < .004), knowledge that the HPV vaccine does not affect growth (78.9% vs 62.7%, OR = 2.23, 95% CI = 1.45–3.42, *p* < .001), perception that the vaccine could protect their daughter (94.3% vs 81.3%, OR = 3.80, 95% CI = 2.12–6.80, *p* < .001), having a doctor recommended the vaccine (11.1% vs 3.8%, OR = 3.17, 95% CI = 1.43–8.78, *p* = .03), and preference for their daughter to receive the vaccine at school (58.8% vs 46.6%, OR = 1.64, 95% CI = 1.09–2.46, *p* = .02) were factors associated with the uptake of HPV vaccine. Conversely, parents who had never heard of the HPV vaccine (1.2% v 4.2%, OR = .28, 95% CI = .10–.81, *p* = .02) and who had a regular family doctor (36.3% vs 47.7%, OR = .63, 95% CI = .42-.93, *p* = .02) were associated with a lower uptake of HPV vaccine at school. In multivariate regression analysis, the perception that the HPV vaccine could protect their daughter from getting cervical cancer showed a 3.16 odds (95% CI = 1.39–7.15, *p* = .006) of joining the HPV vaccination program. In addition, having had doctor’s recommendation showed a 4.54 odds for joining the program (95% CI = 1.05–19.57, *p* = .04), whereas parents of girls who had never heard of HPV vaccine (OR = .15, 95% CI = .03–.71, *p* = .02), parents who were willing to pay more than HK$2000 for the vaccine (OR = .39, 95% CI = .19–.81, *p* = .01) and parents who preferred for their daughter to receive the vaccine at a private clinic (OR = .44, 95% CI = .26 –.75, *p* = .002) were significantly to have lower uptake.

**Table 5:** Analyses of factors associated with parents’ decision-making using multivariate logistic regression (*n* = 857).

	OR	95% CI	*p*
**Never heard of HPV**	.15	.03-.71	.02*
**Correct overall cervical cancer knowledge**	.92	.67–1.26	.60
Cervical cancer is common	1.38	.57–3.32	.48
Early stage cervical cancer is treatable	1.09	.46–2.62	.84
Cervical cancer may affect fertility	1.38	.62–3.09	.43
Cervical cancer is caused by viral infection	0.64	.28–1.42	.27
A woman dies of cervical cancer every 3 days	1.74	.90–3.38	.10
**Correct symptoms–waist pain**	0.43	0.17–1.06	.07
**Correct symptoms–bleeding after sexual intercourse**	1.28	.71–2.32	.42
**Correct Overall HPV vaccine knowledge**	1.07	.50–2.28	.86
HPV vaccine prevents all STIs	.49	.16–1.50	.21
HPV vaccine would not affect growth	1.82	.95–3.51	.07
Females who have had sex can still receive HPV vaccine to prevent future infection	1.42	.73–2.76	.31
**Getting cervical cancer will affect my daughter tremendously**	2.03	.76–5.40	.16
**HPV Vaccine can protect my daughter from HPV infection**	3.16	1.39–7.15	.006**
**Doctor recommended**	4.54	1.05–19.57	.04*
**Has a regular family doctor**	.75	.43–1.30	.30
**Willing to pay more than HK$2,000 for HPV vaccine**	.39	.19-.81	.01*
**Preferred location—private clinics**	.44	.26-.75	.002**
**Preferred location—schools**	1.44	.85–2.45	.17
**Gender–female**	.41	.12–1.37	.15
**Age– 40 or above**	.77	.45–1.61	.33
**Monthly household income–above HK$30,000**	.56	.31–1.02	.06

* = significant at *p*

** = significant at *p*< .01.

### Participation factors among students

Students’ responses were compared based on whether or not they joined the HPV vaccination program and shown in Table 6. The percentage of girls who reported obtaining information on the HPV vaccine from their parents was shown to be significantly higher among students who joined the program (42.6%) than those who did not (28.3%). Moreover, significantly more students in the “joining” group correctly indicated that cervical cancer was one of the most common cancers among women in Hong Kong (82.5% vs. 74.8% in “not joining” group). A significantly higher percentage of students in the “joining” group answered correctly on questions about the HPV vaccine and indicated that it could prevent cervical cancer (88.0%) compared to those in the “not joining” group (63.4%).

**Table 6:** Comparison between students who participated and who did not participate in the program (univariate regressions).

	Joined program		OR	95% CI	*p*
	No *n*(%)	Yes *n*(%)			
**HPV vaccine information source (*n* = 978)**					
Doctor	37 (32.7%)	345 (39.5%)	1.34	.88–2.03	.17
School	52 (46.0%)	466 (53.3%)	1.34	.90–1.99	.15
Parents	32 (28.3%)	372 (42.6%)	1.88	1.22–2.89	.004**
Media	5 (4.4%)	39 (4.5%)	1.01	.39–2.62	.99
Friends / relatives	20 (17.7%)	167 (19.1%)	1.10	.66–1.83	.72
Never heard of HPV	11 (9.7%)	77 (8.8%)	.90	.46–1.74	.75
**Correct cervical cancer knowledge**					
It is a fatal disease (*n* = 1,018)	79 (71.8%)	697 (76.8%)	1.30	.83–2.02	.25
Early stage of cervical cancer is treatable (*n* = 1,018)	64 (56.6%)	596 (65.9%)	1.48	.99–2.20	.05
It is one of the common cancers found in women in HK (*n* = 1,020)	83 (74.8%)	750 (82.5%)	1.59	1.00–2.52	.048*
HPV vaccine can prevent cervical cancer (*n* = 1,019)	71 (63.4%)	798 (88.0%)	4.23	2.74–6.52	<.001***
Females still need to have Pap’s smears regularly after HPV vaccine (*n* = 1,017)	58 (51.8%)	473 (52.3%)	1.02	.69–1.51	.92
**Risks factors of cervical cancer (*n* = 1,030)**					
Smoking is a risk factor	37 (32.5%)	375 (41.0%)	1.45	.96–2.19	.08
Having multiple sex partners is a risk factor	80 (69.6%)	575 (62.8%)	.74	.49–1.13	.16
Not enough sleep is a risk factor	20 (17.4%)	136 (14.9%)	.83	.50–1.39	.48
Prior pregnancy is a risk factor	30 (26.1%)	258 (28.2%)	1.11	.72–1.73	.64
Started sex early is a risk factor	60 (52.2%)	542 (59.2%)	1.33	.90–1.97	.15
**Correct HPV vaccine knowledge (*n* = 1,002)**	82 (75.9%)	756 (84.6%)	1.74	1.08–2.80	.02*
**Cervical cancer more severe than chicken pox (*n* = 937)**	77 (74.0%)	635 (75.8%)	1.10	.69–1.75	.70
**Cervical cancer more severe than hepatitis (*n* = 938)**	40 (38.5%)	334 (40.0%)	1.07	.70–1.62	.76
**Cervical cancer more severe than influenza (*n* = 944)**	62 (60.8%)	553 (65.7%)	1.24	.81–1.88	.33

* = significant at *p*

** = significant at *p*< .01.

*** = significant at p

Univariate logistic regressions were performed to explore the association between students’ knowledge and participation in the vaccination program (Table 6). Results showed that having knowledge of cervical cancer (i.e. one of the common cancers among females in Hong Kong; 82.5% vs 74.8%, OR = 1.59, 95% CI = 1.00–2.52, *p* = .048), obtaining information about the HPV vaccine from their parents (42.6% vs 28.3%, OR = 1.88, 95% CI = 1.22–2.89, *p* = .004), and having correct knowledge of the HPV vaccine (84.6% vs 75.9%, OR = 1.74, 95% CI = 1.08–2.80, *p* = .02) were associated with the uptake of the HPV vaccine. In particular, students who indicated that the HPV vaccine could prevent cervical cancer had an odds ratio of 4.23 (88.0% vs 63.4%, 95% CI = 2.74–6.52, *p* <.001) likelihood of participating in the program. Multivariate logistic regression (Table 7) revealed similar results that parents as the source of HPV vaccine knowledge (OR = 1.97, 95% CI = 1.22–3.16, *p* = .005) and knowledge that the HPV vaccine could prevent cervical cancer (OR = 3.94, 95% CI = 2.35–6.60, *p* <.001) were independent factors significantly associated with increased participation in the program. However, students who perceived that having multiple sex partners was a risk factor for cervical cancer had a lower likelihood of joining the program (OR = .51, 95% CI = .31–.84, *p* = .009).

**Table 7:** Analyses of students’ factors associated with joining the program using multivariate logistic regression (*n* = 928).

	OR	95% CI	*p*
**HPV vaccine source of information**			
Doctor	1.27	.79–2.04	.32
School	1.22	.78–1.90	.39
Parents	1.97	1.22–3.16	.005*
**Knowledge about cervical cancer**			
Early stage of cervical cancer is treatable	1.16	.73–1.85	.54
It is one of the common cancers found in women in HK	1.30	.75–2.23	.35
HPV vaccine can prevent cervical cancer	3.94	2.35–6.60	<0.001**
**Risk factors of cervical cancer**			
Smoking is a risk factor	1.62	.10–2.64	.052
Having multiple sex partners is a risk factor	.51	.31-.84	.009*
Started sex early is a risk factor	1.60	1.00–2.54	.050
**HPV vaccine knowledge**	1.24	.73–2.11	.43

* = significant at *p*< .01.

** = significant at p

## Discussion

This is one of the first studies to investigate the feasibility and acceptability of a subsidized school-based HPV vaccination program in a region without a universal vaccination program. The findings showed that it is possible to deliver a 2-dose HPV vaccination schedule to girls aged between 9 and 14 years in a school setting with an overall uptake rate as high as 80% across the schools.

Prior studies have shown that the uptake rate of the HPV vaccine among females in Hong Kong is low, 7–9% among school-aged girls and 9.7% for university students, as compared to 70% in countries with publicly funded, school-based vaccination programs. The high uptake rates are consistent with results from a study conducted in Hong Kong [12] and comparable to other school-based vaccination programs in Australia and the UK [15, 16]. The improved uptake rates of this study suggests that a school-based approach can address certain barriers and increase likelihood to join the program.

Factors influencing familial decision regarding HPV vaccination include attitudes and recommendations of healthcare providers, beliefs and attitudes of parents and adolescents, peer norms, and communication between parents and adolescents on sexual issues [17]. In our study, it was shown that both parents and students rated vaccine safety and effectiveness as important and influential factors in their decision-making. Among parents who chose not to vaccinate their daughters, the results also showed that concerns about the possible side effects and effectiveness of the vaccine were barriers to acceptance. In contrast to an earlier study which showed that the HPV vaccine may be seen as personally stigmatizing [18] or other negative attitudes, such as concerns around sexual promiscuity, over 80% of parents in this study indicated that the link between HPV vaccination and promiscuity were not a concern. The majority of parents were not worried about the perception of their daughters being sexually active at an early age nor were they concerned that the vaccine would encourage their daughter to begin having sex earlier. Since students must seek parental consent to join the program, it is important to provide accurate information that addresses vaccine safety and effectiveness to parents as well as students.

On the other hand, the views and emotions of adolescents should also be considered. Some students who did not join the program reported that fear of pain from injection was an extremely influential factor to them, which in turn may affect their parents’ decision. It is possible that by conducting the vaccination in a familiar environment, such as in a school setting with their friends and teachers, could reduce fears around vaccination. During vaccination days, we observed many students supporting one another in spite of the fact that many of them were afraid of pain from injection. In addition, regression analysis also showed that students who received information about the HPV vaccine from their parents and perceived that the vaccine could prevent cervical cancer were more likely to participate in the program. Girls with accurate knowledge about cervical cancer, e.g. starting sex at early age as risk factor were likely to participate in the program but knowledge that having multiple sex partners was a risk factor had lower likelihood to join. It is possible that those students did not believe that the risk factor was relevant to them or HPV vaccine might not be protective for those with multiple risk factors.

In places where the HPV vaccination is not subsidized by government, cost has been identified as a crucial factor in many studies [12, 19]. There has been evidence showing that a subsidized program would help increase vaccination uptake among parents. The current market price for two doses of the HPV vaccine in Hong Kong is approximately US$250. However, 64.5% of parents in the current study reported that they would pay, at most, US$125 for the vaccine, so subsiding the cost of the vaccines could address financial barriers and improve the uptake rate. Our results also showed that families with lower income levels were more likely to join the program. Families with higher income levels were less likely to join and preferred to have the vaccine delivered at a private clinic. This suggests that a school-based program could have higher uptake at schools or in areas with lower income families. A school-based model also removes other potential barriers for parents such as having to bring their child to a clinic for vaccination. If a subsidy is offered to students in a school setting, it should be offered equally to all students, regardless of family income to avoid creating negative stigma around receiving the vaccine.

Education and information about cervical cancer and HPV prevention should be tailored around the specific needs of the intended audience, in this case, parents and adolescent female students. For students, having correct knowledge about cervical cancer and HPV prevention did not often translate to participation in the program. Materials should highlight the importance of having the vaccine and encourage students to speak to their parents about it. As an example, students who knew that that having multiple sex partners was a risk factor were less likely to join the program. Educational materials should focus on concerns or information that is more relevant to them.

As students require parental consent to receive the vaccine, providing accurate information and knowledge to parents is crucial. Educational materials for parents should contain clear messages around the safety and effectiveness of the vaccine and address concerns around side effects. During the pilot phase of the project, many parents contacted program staff and teachers with questions around safety of the vaccine. To address their concerns, educational materials were revised to specifically address common questions and misconceptions. In addition, a phone line was established so that parents could call and speak to a healthcare provider directly about their questions. Teachers also received education and informational materials in advance so that they could accurately respond to questions from parents.

A school-based HPV vaccination delivery strategy could be particularly successful in supporting families with lower socio-economic status and result in higher uptake. In October 2016, the Hong Kong Government launched a 3-year HK$98.75M pilot program offering free HPV vaccines to low income families that is expected to benefit 31,000 girls and be delivered through publicly-funded clinics. Our program cost HK$1.4M and benefited over 1,200 girls in both the pilot and final phases of the program. Thus, it represents a lower cost per client when compared to the government pilot program. It is recommended that government also consider piloting larger scale school-based programs in low income areas to assess the uptake rate and effectiveness among different models of delivery.

There are several limitations to this study. First, this is a cross-sectional study which only identifies associating factors rather than causation. Secondly, there may be self-selection bias as the participation of schools, students and parents was voluntary, which could affect the representativeness of our study population. Our study population consisted of schools with higher proportion of families from lower socio-economic income levels and results should be interpreted with caution. Nevertheless, our study removed the financial barriers by providing the vaccine free of cost, which allowed us to develop a clearer understanding of the other facilitators and barriers to vaccination among students and parents.

## Conclusions

Approximately half of the cervical cancer cases globally are in Asia, but currently only a few large-scale HPV vaccination programs have been implemented in the region, such as Macau and South Korea. In places without such programs like Hong Kong, Singapore, or Taiwan, the uptake rate of conventional vaccine-preventable diseases, such as measles, is high while the uptake of HPV vaccine remains low. The current study provides empirical data that supports the feasibility of a school-based HPV vaccination program as a vaccine delivery model that can significantly improve uptake in Hong Kong.

## Supporting information

S1 File Questionnaire for parents in Chinese and English. (PDF)

S2 File Questionnaire for students in Chinese and English. (PDF)

## References

[1] Department of Health. Cervical cancer screening coverage Hong Kong2016 [cited 2017 June]. Available from: http://www.cervicalscreening.gov.hk/english/sr/sr_statistics_ccsc.html.

[2] Choi HC, Leung GM, Woo PP, Jit M, Wu JT. Acceptability and uptake of female adolescent HPV vaccination in Hong Kong: A survey of mothers and adolescents. Vaccine. 2013;32(1):78–84. doi: 10.1016/j.vaccine.2013.10.068 24188759 10.1016/j.vaccine.2013.10.068

[3] Lee A, Ho M, Cheung CKM, Keung VMW. Factors influencing adolescent girls’ decision in initiation for human papillomavirus vaccination: a cross-sectional study in Hong Kong. BMC public health. 2014;14(1):925. 25195604 10.1186/1471-2458-14-925 PMC4176578

[4] Li SL, Lau YL, Lam TH, Yip PSF, Fan SYS, Ip P. HPV vaccination in Hong Kong: Uptake and reasons for non-vaccination amongst Chinese adolescent girls. Vaccine. 2013;31(49):5785–8. doi: 10.1016/j.vaccine.2013.10.027 24148571 10.1016/j.vaccine.2013.10.027

[5] Chen JM, Leung DY. Factors Associated with Human Papillomavirus Vaccination among Chinese Female University Students in Hong Kong. Am Int J Soc Sci. 2016;3:56–62.

[6] Becker MH. The health belief model and personal health behavior. Health Education Monographs. 1974;2:324–473.

[7] Fishbein M, editor A theory of reasoned action: some applications and implications. Nebraska Symposium on Motivation Nebraska Symposium on Motivation; 1980. 7242751

[8] Rosenstock IM. The health belief model and preventive health behavior. Health education monographs. 1974;2(4):354–86. 10.1177/109019817800600406 299611

[9] Katz IT, Ware NC, Gray G, Haberer JE, Mellins CA, Bangsberg DR. Scaling up human papillomavirus vaccination: a conceptual framework of vaccine adherence. Sexual health. 2010;7(3):279–86. doi: 10.1071/SH09130 20719215 10.1071/SH09130 PMC3141556

[10] Kwan TT, Chan KK, Yip AM, Tam K, Cheung AN, Lee P, et al Barriers and facilitators to human papillomavirus vaccination among Chinese adolescent girls in Hong Kong: a qualitative–quantitative study. Sexually transmitted infections. 2008;84(3):227–32. doi: 10.1136/sti.2007.029363 18256106 10.1136/sti.2007.029363

[11] Kessels SJ, Marshall HS, Watson M, Braunack-Mayer AJ, Reuzel R, Tooher RL. Factors associated with HPV vaccine uptake in teenage girls: a systematic review. Vaccine. 2012;30(24):3546–56. doi: 10.1016/j.vaccine.2012.03.063 22480928 10.1016/j.vaccine.2012.03.063

[12] Lee A, Wong MC, Chan TT, Chan PK. A home-school-doctor model to break the barriers for uptake of human papillomavirus vaccine. BMC public health. 2015;15(1):935. 26392084 10.1186/s12889-015-2269-1 PMC4578840

[13] Lee A, Chan P, Lau L, Chan T. How would family physicians facilitate the uptake of HPV vaccination: focus group study on parents and single women in Hong Kong. Hong Kong Practitioner. 2011;33(3):07–114.

[14] Information Services Department. Hong Kong: The facts Hong Kong: Information Services Department HKSAR; 2015. Available from: https://www.gov.hk/en/about/abouthk/factsheets/docs/population.pdf.

[15] Brabin L, Roberts SA, Stretch R, Baxter D, Chambers G, Kitchener H, et al Uptake of first two doses of human papillomavirus vaccine by adolescent schoolgirls in Manchester: prospective cohort study. Bmj. 2008;336(7652):1056–8. doi: 10.1136/bmj.39541.534109.BE 18436917 10.1136/bmj.39541.534109.BE PMC2375997

[16] Watson M, Shaw D, Molchanoff L, McInnes C. Challenges, lessons learned and results following the implementation of a human papilloma virus school vaccination program in South Australia. Australian and New Zealand journal of public health. 2009;33(4):365–70. doi: 10.1111/j.1753-6405.2009.00409.x 19689598 10.1111/j.1753-6405.2009.00409.x

[17] Gamble HL, Klosky JL, Parra GR, Randolph ME. Factors influencing familial decision-making regarding human papillomavirus vaccination. Journal of Pediatric Psychology. 2009;35(7):704–15. doi: 10.1093/jpepsy/jsp108 19966315 10.1093/jpepsy/jsp108 PMC2915623

[18] Lee PW, Kwan TT, Tam KF, Chan KK, Young PM, Lo SS, et al Beliefs about cervical cancer and human papillomavirus (HPV) and acceptability of HPV vaccination among Chinese women in Hong Kong. Preventive medicine. 2007;45(2):130–4. 17707077 10.1016/j.ypmed.2007.07.013

[19] Leask J, Chapman S, Hawe P, Burgess M. What maintains parental support for vaccination when challenged by anti-vaccination messages? A qualitative study. Vaccine. 2006;24(49):7238–45. 17052810 10.1016/j.vaccine.2006.05.010

