# Supplementary material for: Uptake of human papillomavirus (HPV) vaccination in Hong Kong: Facilitators and barriers among adolescent girls and their parents
Source: PLoS One. 2018 Mar 15;13(3):e0194159. doi: 10.1371/journal.pone.0194159 (PMC5854366; doi:10.1371/journal.pone.0194159)
Supplement: S1 File — (PDF) [file pone.0194159.s001.pdf]

香港中文大學
健康教育及促進健康中心和微生物學系
預防子宮頸癌研究計劃

家長問卷調查

香港中文大學健康教育及促進健康中心和香港中文大學微生物學系正進行一項有關預防子宮頸癌的研究，探討大眾對疾病預防的認知。現誠邀**你及你的女兒每人填寫一份調查問卷，合共兩份調查問卷**，以協助我們計劃日後公共衛生教育的推廣和發展。

本問卷以不記名形式進行，你的身份及所提供的資料將會絕對保密，並只作研究用途。

****

請細閱以下各題，並在合適的方格內填上「✔」。

1. 你會否讓女兒參與是次「預防子宮頸癌疫苗接種計劃」？

☐ 會 → 請答 **1A** 及 第 **2** 題

☐ 不會 → 請答 **1B** 及 第 **2** 題

☐ 女兒之前已接種預防子宮頸癌疫苗 → 請到第 **2** 題

**1A.** 以下因素如何影響你讓女兒參與是次「預防子宮頸癌疫苗接種計劃」？	絕對沒有影響	沒有影響	有影響	很大影響
a. 是次計劃由校本統籌	☐	☐	☐	☐
b. 是次計劃由大學推薦	☐	☐	☐	☐
c. 疫苗能有效預防子宮頸癌	☐	☐	☐	☐
d. 疫苗安全	☐	☐	☐	☐
e. 免費接種疫苗	☐	☐	☐	☐
f. 醫生建議	☐	☐	☐	☐

**1B.** 以下因素如何影響你 <u>不讓</u> 女兒參與是次「預防子宮頸癌疫苗接種計劃」？	絕對沒有影響	沒有影響	有影響	很大影響
a. 注射疫苗後的副作用	☐	☐	☐	☐
b. 不相信疫苗的成效	☐	☐	☐	☐
c. 醫生沒有建議	☐	☐	☐	☐
d. 政府沒有建議	☐	☐	☐	☐
e. 擔心女兒接種疫苗後，別人誤以為她已開始了性行為	☐	☐	☐	☐
f. 擔心女兒接種疫苗後，會提早發生性行為	☐	☐	☐	☐

2. 你從甚麼途徑認識預防子宮頸癌疫苗？（可選多項）

☐ 醫生推薦 ☐ 學校
☐ 診所內的海報／宣傳單張 ☐ 傳媒資訊（包括電視、電台、報章、雜誌、互聯網等）
☐ 親戚／朋友推薦 ☐ 沒有聽過這種疫苗

3. 以下各項對子宮頸癌的描述是否正確？

		是	不是	不知道
a.	子宮頸癌是其中一種香港女性普遍出現的癌症。	☐	☐	☐
b.	子宮頸癌及早發現是容易醫治的。	☐	☐	☐
c.	婦女患子宮頸癌後，可能會影響生育。	☐	☐	☐
d.	女性如有多位性伴侶，會增加患上子宮頸癌的機會。	☐	☐	☐
e.	只要有性生活的女士，就有機會患上子宮頸癌。	☐	☐	☐
f.	接受柏氏子宮頸抹片檢查可以預防子宮頸癌。	☐	☐	☐
g.	子宮頸癌是由病毒感染引起。	☐	☐	☐
h.	在香港，大約每三日有一位女士死於子宮頸癌。	☐	☐	☐

4. 以下哪些是最常見的子宮頸癌病徵？（可選多項）

☐ 腰痛 ☐ 怕冷 ☐ 兩次經期間不正常出血
☐ 胸部脹痛 ☐ 性行為後陰道出血

5. 請從以下選出對預防子宮頸癌疫苗的正確描述：（可選多項）

☐ 少女未有初次月經前，不適合注射預防子宮頸癌疫苗
☐ 接種子宮頸癌疫苗的最佳時期是未曾有性行為之前
☐ 接種子宮頸癌疫苗後，能預防感染所有性病
☐ 注射預防子宮頸癌疫苗，與月經無關，亦不會影響發育
☐ 已有性行為的女性，接種子宮頸癌疫苗，亦可為未來提供保護

**6.**	你認為以下各項的可能性如何？	極不可能	不可能	可能	極可能
a.	你認為女兒會感染人類乳頭瘤病毒 (HPV) 嗎？	☐	☐	☐	☐
b.	你認為女兒會患上子宮頸癌嗎？	☐	☐	☐	☐
c.	一旦患上子宮頸癌，會為女兒的生活帶來很大的影響嗎？	☐	☐	☐	☐
d.	接受預防子宮頸癌疫苗注射，能為女兒提供保護，避免染上 HPV 病毒嗎？	☐	☐	☐	☐

7. 醫生曾否建議你的女兒接受預防子宮頸癌疫苗注射？

☐ 有
☐ 沒有 →**請到第 9 題**

8. 接上題：如有的話，醫生認為你的女兒甚麼時候需要接種？

☐ 現階段有需要 ☐ ______ 歲 （請填歲數）之後 ☐ 醫生沒有說明

9. 一般來說，你會聽從醫生有關預防疫苗的建議嗎？

☐ 多數會 ☐ 視乎個別情況 ☐ 不會

10. 你的性別是： ☐ 男 ☐ 女

11. 你與學童的關係是：

☐ 父 / 母 ☐ 兄 / 姊 ☐ 親戚 ☐ 其他（請說明：____________________）

12. 你的年齡是：

☐ 30 歲或以下 ☐ 31-35 歲 ☐ 36-40 歲

☐ 41-45 歲 ☐ 46-50 歲 ☐ 51 歲或以上

13. 你的教育程度是：

☐ 小學或以下 ☐ 中學 ☐ 大專 ☐ 大學或以上

14. 你的家庭每月總收入（以港幣計算）：

☐ $5,000 或以下

☐ $5,001- $10,000

☐ $10,001- $15,000

☐ $15,001- $20,000

☐ $20,001- $25,000

☐ $25,001- $30,000

☐ $30,001 或以上

15. 你有沒有一位固定的家庭醫生？

☐ 有 ☐ 沒有

16. 你的女兒是否已接受所有香港衛生署建議的疫苗注射？

☐ 是 ☐ 不是

17. 你／你的太太在過去三年，有沒有接受過柏氏子宮頸抹片檢查？

☐ 有 ☐ 沒有

18. 你／你的太太有沒有接種預防子宮頸癌疫苗？

☐ 有 ☐ 沒有

19. 你願意支付多少錢，讓女兒接種子宮頸癌疫苗（整個療程並以港幣計算）？

☐ 少於 1000 元 ☐ 1001 至 1500 元 ☐ 1501 至 2000 元

☐ 2001 至 2500 元 ☐ 2501 至 3000 元 ☐ 3001 至 3500 元

我們亦希望收集你的意見，作為日後讓政府考慮推行全民接種子宮頸癌預防疫苗之用。
請你回答以下各題：

20. 假如政府推行全民接種子宮頸癌預防疫苗，你贊成同時讓男女學生參與嗎？
- ☐ 贊成同時讓男女學生參與
- ☐ 只讓女學生參與

21. 假如推行全民接種計劃，你認為接受子宮頸癌疫苗（以整個療程計算），政府在價錢上應提供多少資助？
- ☐ 全部免費
- ☐ 政府提供百分之 ______（請填上百分比）的資助

22. 假如政府推行全民接種計劃，你認為哪裡是最合適的接種地點？（可選多項）
- ☐ 衞生署指定健康中心
- ☐ 私家醫生診所
- ☐ 學校

23. 你認為接種子宮頸癌預防疫苗最好由幾時開始？
- ☐ 9 歲
- ☐ 小學六年級
- ☐ 中學一年級
- ☐ 中學三年級
- ☐ 按照政府指示

24. 你認為自己對子宮頸癌預防疫苗的認識是否足夠？
- ☐ 足夠
- ☐ 不足夠，並希望獲得更多以下資訊（可選多項）：
  - ☐ 對子宮頸癌的認識
  - ☐ 對預防子宮頸癌疫苗的認識
  - ☐ 疫苗的效益
  - ☐ 疫苗的副作用
  - ☐ 醫生／專家的意見

25. 你有否看了學校通告提及有關子宮頸癌的錄影片段？

☐ 有　☐ 沒有　☐ 沒留意　☐ 已經忘記了內容

26. 如有，會否影響你**讓**女兒參與是次「預防子宮頸癌疫苗接種計劃」？

**絕對沒有影響**	**沒有影響**	**有影響**	**很大影響**
☐	☐	☐	☐

問卷完，謝謝！

The Chinese University of Hong Kong
Centre for Health Education and Health Promotion and Department of Microbiology
Prevention of Cervical Cancer Vaccination Programme

Questionnaire – Parent

Please read the following questions and put a ✓ in the box next to your choice.

1. Are you going to let your daughter join the "Prevention of Cervical Cancer Vaccination Programme"?

- ☐ Yes → Please answer Q1A and Q2
- ☐ No → Please answer Q1B and Q2
- ☐ My daughter has already received HPV vaccination → Please go to Q2

**1A.** How the following factors affect your decision to let your daughter join the programme?	Not at all Influential	Not influential	Influential	Extremely Influential
a. This programme is organized by school	☐	☐	☐	☐
b. This programme is recommended by the university	☐	☐	☐	☐
c. Vaccine provides effective prevention of cervical cancer	☐	☐	☐	☐
d. Vaccine is safe	☐	☐	☐	☐
e. Free vaccination	☐	☐	☐	☐
f. Recommended by doctor(s)	☐	☐	☐	☐

**1B.** How the following factors affect your decision NOT to let your daughter join the programme?	Not at all Influential	Not influential	Influential	Extremely Influential
a. Possible side effect	☐	☐	☐	☐
b. Doubt the effectiveness of vaccine	☐	☐	☐	☐
c. No doctor's recommendation	☐	☐	☐	☐
d. No government's recommendation	☐	☐	☐	☐
e. Worry others might think my daughter has already started having sex if vaccinated	☐	☐	☐	☐
f. Worry that my daughter would start having sex earlier if vaccinated	☐	☐	☐	☐

2. How did you learn about HPV vaccination? (You may choose more than one answer)

- ☐ Doctor's recommendation
- ☐ School
- ☐ Posters/leaflets in clinic
- ☐ Mass media (including television, radio, newspapers, magazines and internet etc.)
- ☐ Relatives / Friends
- ☐ Never heard of HPV vaccination

**3.** Are the following statements about cervical cancer correct?	Yes	No	Don't Know
a. Cervical cancer is one of the most common cancers among women in Hong Kong.	☐	☐	☐
b. Early stage of cervical cancer is easy to treat.	☐	☐	☐
c. Females who have got cervical cancer may affect fertility.	☐	☐	☐
d. Females who have multiple sex partners are having a higher risk of getting cervical cancer.	☐	☐	☐
e. Any females who have sexual experience may have a fair chance of getting cervical cancer.	☐	☐	☐
f. Cervical cancer can be prevented through Pap's smear.	☐	☐	☐
g. Cervical cancer is caused by viral infection.	☐	☐	☐
h. In Hong Kong, about a woman died of cervical cancer every 3 days.	☐	☐	☐

4. Which of the following(s) is(are) the most common symptoms of cervical cancer? (You may choose more than one answer)

☐ Waist pain ☐ Fear of cold ☐ Abnormal bleeding between menses

☐ Breast pain ☐ Bleeding after sexual intercourse

5. Which of the following(s) is/are correct description of HPV vaccination? (You may choose more than one answer)

☐ Girls should not receive HPV vaccination before their first menstruation.

☐ The most ideal time to have HPV vaccination is before first sexual intercourse.

☐ HPV vaccination can prevent all sexually transmitted disease.

☐ HPV vaccination is not related to menstruation and would not affect growth.

☐ Females already started sexual life can still receive HPV vaccination to prevent future infection.

**6.** What do you think about the likelihood of the followings?	Extremely unlikely	Unlikely	Likely	Extremely Likely
a. My daughter will be infected by Human Papillomavirus (HPV).	☐	☐	☐	☐
b. My daughter will get cervical cancer.	☐	☐	☐	☐
c. It will bring tremendous influence to my daughter's life if she got cervical cancer.	☐	☐	☐	☐
d. HPV vaccination can protect my daughter from HPV infection.	☐	☐	☐	☐

7. Did doctor recommend your daughter to take HPV vaccination?

☐ Yes

☐ No → Please go to Q9

8. From your doctor's recommendation, what is the best age for your daughter to get HPV vaccination?

☐ Now ☐ After age _______ ☐ Doctor did not mention

9. General speaking, would you follow doctor's advice on vaccination in preventing diseases?

☐ No ☐ Depends on the situation ☐ Probably yes

10. What is your gender： ☐ Male ☐ Female

11. What is your relationship with the student?

☐ Father / mother ☐ Brother / sister ☐ Relative ☐ Other, please specify: ______

12. What is your age?

☐ Below 30 ☐ 31-35 ☐ 36-40
☐ 41-45 ☐ 46-50 ☐ 51 or above

13. . What is the highest level of education you have completed?

☐Primary or below ☐Secondary School ☐ Post-secondary/Diploma ☐Degree or higher

14. What is your family income per month?

☐ $5,000 or below ☐ $5,001- $10,000 ☐ $10,001- $15,000 ☐ $15,001- $20,000
☐ $20,001- $25,000 ☐ $25,001- $30,000 ☐ $30,001 or above

15. Do you have a regular family doctor?

☐ Yes ☐ No

16. Have your daughter received all vaccination recommendation by Department of Health?

☐ Yes ☐ No

17. Have you / your wife undergone Pap's smear in the past 3 years?

☐ Yes ☐ No

18. Have you / your wife received HPV vaccination?

☐ Yes ☐ No

19. How much are you willing to pay for daughter's HPV vaccination (whole course in HKD)?

☐ Less than $1000 ☐ $1001-$1500 ☐ $1501-$2000
☐ $2001- 2500 ☐ $2501–3000 ☐ $3001-3500

We would also like to know what you think about including HPV vaccination into government's childhood immunization programme in Hong Kong. Please answer the following questions:

20. If HPV vaccination is included in the government's childhood immunization programme, do you think it should be provided for both boys and girls?

☐ Yes
☐ Only girls should join

21. If HPV vaccination is included in the government's childhood immunization programme, how much should the government subsidize?

- ☐ Totally Free
- ☐ Government subsidizes _______ % (Please fill in percentage)

22. If HPV vaccination is included in the government's childhood immunization programme, where is/are ideal vaccination location(s)? (You may choose more than one answer)

- ☐ Health centres under Department of Health
- ☐ Private physicians' clinics
- ☐ Schools

23. When do you think is the best time to have HPV vaccination?

☐ Age of 9 ☐ Primary 6 ☐ Secondary 1

☐ Secondary 3 ☐ According to government's instruction

24. Do you think you have sufficient understanding of HPV vaccination?

- ☐ Yes
- ☐ No, and would like to have more information on the following(s) (may choose more than 1 answer)
  - ☐ Knowledge on cervical cancer
  - ☐ Knowledge on HPV vaccine
  - ☐ Effectiveness of HPV vaccine
  - ☐ Possible side effects
  - ☐ Doctors'/Experts' recommendation

25. Have you watched the video on cervical cancer mentioned in the school notice?

☐ Yes ☐ No ☐ Not Aware of it ☐ Forgot the contents

**26.** If yes, how does it affect your decision to let your daughter join the programme?	Not at all Influential	Not influential	Influential	Extremely Influential
	☐	☐	☐	☐

End of Questionnaire. Thank you very much!
