# Supplementary material for: Uptake of human papillomavirus (HPV) vaccination in Hong Kong: Facilitators and barriers among adolescent girls and their parents
Source: PLoS One. 2018 Mar 15;13(3):e0194159. doi: 10.1371/journal.pone.0194159 (PMC5854366; doi:10.1371/journal.pone.0194159)
Supplement: S2 File — (PDF) [file pone.0194159.s002.pdf]

本問卷供學童填寫

香港中文大學
健康教育及促進健康中心和微生物學系
預防子宮頸癌計劃

學童問卷調查

請細閱以下各題，並在合適的方格內填上「✓」。

1. 你的出生日期是：__________年 _________月 ________日

2. 你會否參與是次「預防子宮頸癌疫苗接種計劃」？

☐ 會 → 請答 **2A** 及 第 **3** 題

☐ 不會 → 請答 **2B** 及 第 **3** 題

☐ 本身已接種子宮頸癌預防疫苗 → 請跳到第 **4** 題

2A. 以下因素如何影響你**參與**是次「預防子宮頸癌疫苗接種計劃」？

	**絕對沒有影響**	**沒有影響**	**有影響**	**有很大影響**
a. 是次計劃由校本統籌	☐	☐	☐	☐
b. 是次計劃由大學推薦	☐	☐	☐	☐
c. 疫苗能有效預防子宮頸癌	☐	☐	☐	☐
d. 疫苗安全	☐	☐	☐	☐
e. 免費接種疫苗	☐	☐	☐	☐
f. 醫生建議	☐	☐	☐	☐
g. 父母／家人／親戚建議	☐	☐	☐	☐
h. 同學／朋友一起參與	☐	☐	☐	☐

2B. 以下因素如何影響你<u>不參與</u>是次「預防子宮頸癌疫苗接種計劃」？

	絕對沒有影響	沒有影響	有影響	有很大影響
a. 注射疫苗後的副作用	☐	☐	☐	☐
b. 不相信疫苗的成效	☐	☐	☐	☐
c. 醫生沒有建議	☐	☐	☐	☐
d. 政府沒有建議	☐	☐	☐	☐
e. 父母／家人／親戚反對	☐	☐	☐	☐
f. 同學／朋友不參加	☐	☐	☐	☐
g. 擔心會影響發育	☐	☐	☐	☐
h. 怕痛	☐	☐	☐	☐

3. 接上題，誰人決定你是否參與是次計劃？

☐ 爸爸 / 媽媽

☐ 自己

☐ 哥哥 / 姊姊

☐ 其他，請註明關係：________________________

＜轉後頁＞

4. 你從甚麼途徑認識子宮頸癌預防疫苗？（可選多項）
傳媒資訊（包括電視、電台、報章、雜誌、互聯網等）
☐ 醫生／診所　☐ 父母／家人　☐ 沒有聽過這種疫苗
☐ 親戚／朋友　☐ 學校

5. 請從以下選出 **一項** 對子宮頸癌預防疫苗的正確描述：
☐ 女性未有初次月經前，不適合注射子宮頸癌預防疫苗
☐ 接種子宮頸癌預防疫苗後，能預防感染所有性病
☐ 注射子宮頸癌預防疫苗，與月經無關，亦不會影響發育

6. 以下哪些因素，會增加患上子宮頸癌的機會？（可選多項）
☐ 吸煙　☐ 有多位性伴侶　☐ 睡眠不足
☐ 曾懷孕　☐ 早發生性行為

以下各題對子宮頸癌的描述是否正確？	**是**	**不是**	**不知道**
**7.** 子宮頸癌是致命性的疾病	☐	☐	☐
**8.** 子宮頸癌是其中一種香港女性普遍出現的癌症	☐	☐	☐
**9.** 接種子宮頸癌預防疫苗，是有效的預防方法	☐	☐	☐
**10.** 在接種子宮頸癌預防疫苗後，都需要定期接受子宮頸檢查	☐	☐	☐
**11.** 子宮頸癌及早發現是容易醫治的	☐	☐	☐

12. 你有否看了學校通告提及有關子宮頸癌的錄影片段？
☐ 有　☐ 沒有　☐ 沒留意　☐ 已經忘記了內容

	**絕對沒有影響**	**沒有影響**	**有影響**	**很大影響**
**13.** 如有，會否影響你參與是次「預防子宮頸癌疫苗接種計劃」？	☐	☐	☐	☐

14. 你認為以下各種疾病的嚴重性如何？請圈出：以 4 代表最嚴重， 1 代表最不嚴重。

	**最不嚴重**			**最嚴重**	**不知道**
a. 水痘	1	2	3	4	☐
b. 子宮頸癌	1	2	3	4	☐
c. 肝炎	1	2	3	4	☐
d. 流感	1	2	3	4	☐

**13.** 你曾否接種以下預防疫苗：	**有**	**沒有**	**不知道**
a. 水痘疫苗	☐	☐	☐
b. 子宮頸癌疫苗	☐	☐	☐
c. 肺炎球菌疫苗	☐	☐	☐
d. 流感疫苗	☐	☐	☐

問卷完，謝謝！

The Chinese University of Hong Kong
Centre for Health Education and Health Promotion and Department of Microbiology
Prevention of Cervical Cancer Vaccination Programme
Questionnaire – Student

Please read the following questions and put a ✓ in the box next to your choice or write in the space provided.

1. Your Date of Birth： ___________(Year) __________ (Month) _________ (Date)

2. Are you going to join the "Prevention of Cervical Cancer Vaccination Programme"?
   - ☐ Yes → Please answer Q2A and Q3
   - ☐ No → Please answer Q2B and Q3
   - ☐ I have already received HPV vaccination → Please go to Q4

2A. How has the following factors affect your decision to join the programme?	Not at all Influential	Not influential	Influential	Extremely Influential
a. This programme is organized by school	☐	☐	☐	☐
b. This programme is recommended by university	☐	☐	☐	☐
c. Vaccine can effectively prevent cervical cancer	☐	☐	☐	☐
d. Vaccine is safe	☐	☐	☐	☐
e. Free vaccination	☐	☐	☐	☐
f. Doctor's recommendation	☐	☐	☐	☐
g. Parents'/family members' recommendation	☐	☐	☐	☐
h. Classmates/friends join the programme	☐	☐	☐	☐

2A. How do the following factors affect your decision NOT to join the programme?	Not at all Influential	Not influential	Influential	Extremely Influential
a. Possible side effect	☐	☐	☐	☐
b. Doubt the effectiveness of vaccine	☐	☐	☐	☐
c. No doctor's recommendation	☐	☐	☐	☐
d. No government's recommendation	☐	☐	☐	☐
e. Objection from Parents / family members	☐	☐	☐	☐
f. Classmates/friends have not joined	☐	☐	☐	☐
g. Worry that vaccine may affect growth	☐	☐	☐	☐
h. Fear of the pain of injection	☐	☐	☐	☐

3. Who made the decision to let you join the programme?
   - ☐ Father / Mother
   - ☐ Myself
   - ☐ Elder brother / Elder sister
   - ☐ Others, please specify: _________________________

4. How did you learn about HPV vaccination? (You may choose more than one answer)

☐ Mass media (including television, radio, newspapers, magazines and internet, etc.)

☐ Doctor / Clinic ☐ Parents / family members ☐ Never heard of HPV vaccination

☐ Relatives / Friends ☐ School

5. Which one of the following do you think is correct about HPV vaccination?

☐ Girls should not receive HPV vaccination before their first menstruation

☐ HPV vaccination can provide protection to all sexually transmitted disease

☐ HPV vaccination is not related to menstruation and would not affect growth

6. Which of the following(s) increase the likelihood of getting cervical cancer? (may choose >1 answer)

☐ Smoking ☐ Multiple sex partners ☐ Not enough sleep

☐ Pregnant before ☐ Early sex

Are the following statements about cervical cancer correct?	Yes	No	Don't Know
7. Cervical cancer is a fetal disease.	☐	☐	☐
8. Cervical cancer is one of the common cancers found women in Hong Kong.	☐	☐	☐
9. HPV vaccination is an effective way to prevent cervical cancer.	☐	☐	☐
10. Females still need to receive Pap's smears regularly after HPV vaccination	☐	☐	☐
11. Early stage of cervical cancer is easy to treat.	☐	☐	☐

12. Did you watch the video on cervical cancer mentioned in the school notice?

☐ Yes ☐ No ☐ No aware of it ☐ Forgot the contents

13. If yes, how does it affect your decision to join the programme?	Not at all Influential	Not influential	Influential	Extremely influential
	☐	☐	☐	☐

14. What do you think about the severity of the following diseases? Please circle your answer: 1 = Not at all severe , 4= Extremely severe.

	Not at all			Extremely Severe	Don't Know
a. Chicken pox	1	2	3	4	☐
b. Cervical Cancer	1	2	3	4	☐
c. Hepatitis	1	2	3	4	☐
d. Influenza	1	2	3	4	☐

13. Have you ever received any of the following vaccination?

	Yes	No	Don't Know
a. Varicella vaccine	☐	☐	☐
b. HPV vaccine	☐	☐	☐
c. Pneumococcal vaccine	☐	☐	☐
d. Influenza vaccine	☐	☐	☐

End of Questionnaire. Thank You!
